# Serosurveillance of *Coxiella burnetii* in feral swine populations of Hawaiʻi and Texas identifies overlap with human Q fever incidence

**DOI:** 10.1128/jcm.00780-24

**Published:** 2024-08-27

**Authors:** Ian A. McMillan, Michael H. Norris, Samuel J. Golon, Gregory A. Franckowiak, James M. Grinolds, Samuel M. Goldstein, Darrin M. Phelps, Michael J. Bodenchuk, Bruce R. Leland, Richard A. Bowen, Vienna R. Brown, Bradley R. Borlee

**Affiliations:** 1 School of Life Sciences, University of Hawaiʻi at Mānoa, Honolulu, Hawaiʻi, USA; 2 Pathogen Analysis and Translational Health Group, School of Life Sciences, University of Hawaiʻi at Mānoa, Honolulu, Hawaiʻi, USA; 3 Department of Microbiology, Immunology and Pathology, Colorado State University, Fort Collins, Colorado, USA; 4 US Department of Agriculture, Animal and Plant Health Inspection Service, Wildlife Services, National Feral Swine Damage Management Program, Fort Collins, Colorado, USA; 5 US Department of Agriculture, Animal and Plant Health Inspection Service, Wildlife Services, Honolulu, Hawaiʻi, USA; 6 US Department of Agriculture, Animal and Plant Health Inspection Service, Wildlife Services, San Antonio, Texas, USA; 7 Department of Biomedical Sciences, Colorado State University, Fort Collins, Colorado, USA; University of California, Davis, Davis, California, USA

**Keywords:** feral swine, *Coxiella burnetii*, Q fever, seroprevalence

## Abstract

Feral swine are invasive in the United States and a reservoir for infectious diseases. The increase in feral swine population and the geographic range are a concern for the spread of zoonotic diseases to humans and livestock. Feral swine could contribute to the spread of *Coxiella burnetii,* the causative agent of human Q fever. In this study, we characterized the seroprevalence of *C. burnetii* in feral swine populations of Hawaiʻi and Texas, which have low and high rates of human Q fever, respectively. Seropositivity rates were as high as 0.19% and 6.03% in Hawaiʻi and Texas, respectively, indicating that feral swine cannot be ruled out as a potential reservoir for disease transmission and spread. In Texas, we identified the overlap between seropositivity of feral swine and human Q fever incidence. These results indicate that there is a potentially low but detectable risk of *C. burnetii* exposure associated with feral swine populations in Hawaiʻi and Texas.

## INTRODUCTION

Q fever was first observed in abattoir workers in Brisbane, Australia, between the years of 1933 and 1937 (1). Diagnostic techniques available at that time, including blood cultures and agglutination tests, were unable to identify the cause of illness in approximately 20 cases out of 800 employees, leading investigators to query the cause of this fever-inducing agent (1). Today, we know that Q fever is a zoonosis caused by a small coccobacillus, *Coxiella burnetii*, most commonly after exposure to contaminated aerosols (2). *C. burnetii* is a small obligate intracellular pleomorphic rod that has a cell wall structure similar to that of a Gram-negative bacteria (3). Growth of *C. burnetii* includes a biphasic cycle between a small cell variant (SCV) and a large cell variant (LCV). The LCV is considered the replicative form of *C. burnetii* that eventually differentiates into the SCV during stationary phase growth (4, 5). After long-term experimental growth, the majority of *C. burnetii* will be in the SCV form (4, 5). The nonreplicating SCV is highly stable in the environment, resistant to osmotic and physical stress, and equally as infective as the LCV (4–6). The SCV is likely important to ensure environmental stability and continuation of the zoonotic cycle of infection.

During infection, *C. burnetii* infects mononuclear phagocytes and replicates within a phagolysosome-like structure (7–9). Trophoblast cells within the placenta can also become infected, leading to preterm births and fetal death (10, 11). Aborted placental tissue and fluids from infected livestock can contain up to a billion *C. burnetii* organisms per gram and are a major source of environmental contamination linked to outbreaks of human Q fever (10, 12–18). *C. burnetii* has a very low infectious dose, shown to be as low as a single bacterium, that can manifest as an acute self-limiting febrile illness with influenza-like symptoms (12, 19–21). More severe forms of Q fever manifest months to years after acute infection in the form of endocarditis, chronic hepatitis, osteoarticular infections, or pseudo-tumors of the spleen and lung (2, 22–24). While birth products from animals contain the highest concentration of *C. burnetii* and are reported to be the most likely transmission source for human infection*,* other excreted products like urine, feces, and milk have also been shown to harbor the pathogen (2, 25, 26). An increasing list of potential reservoirs have been reported, including marine mammals, domestic mammals, birds, reptiles, and ticks, while some reports also indicate potential epidemiological associations to human Q fever (27–34).

Feral swine are an invasive species introduced into the United States by Spanish explorers in the 1500s and have significantly expanded their range to at least 38 states (35, 36). Swine were introduced to Hawaiʻi much earlier when Polynesian voyagers settled on the islands (37). Feral swine are the same species as domesticated swine, *Sus scrofa*, and often include a variety of phenotypic and genotypic traits from both populations (35, 38). At about 1 year of age, female swine can breed and produce up to ten piglets each year (39). The rapid reproduction rate and lack of predation have aided the geographic and population expansion of feral swine (35). The expansion of feral swine across the United States is a concern for numerous reasons, including conservation of threatened/endangered species; damage to agriculture and livestock, leading to major economic loss; and transmission of pathogens to humans or livestock (40). For example, a proportion of feral swine in the state of Texas have been found to harbor antibodies against *Bacillus anthracis*, suggesting exposure to the causative agent of anthrax (41). Feral swine are also a reservoir for other human and animal diseases including, but not limited to, brucellosis, trichinellosis, tuberculosis, leptospirosis, and classical swine fever (42). Understanding the distribution of pathogens within feral swine populations is critical to identify and mitigate threats to humans, livestock, and wildlife from the pathogens disseminated by this invasive and destructive species.

The role of swine as a potential reservoir of *C. burnetii* is not well-understood. Several groups have investigated domesticated pigs and were unable to exclude swine as a reservoir for human Q fever infections (43–45). Studies in South Korea, China, and Italy have previously identified low but detectable levels of *C. burnetii* seroprevalence in domesticated pigs. Feral swine have also shown low levels of seropositivity toward *C. burnetii* in Portugal, Spain, and South Korea, mirroring the results observed in domesticated pigs (46–48). In Queensland, Australia, a serological survey of pig-hunting dogs identified that 18.3% had antibodies targeting *C. burnetii,* although a direct link of transmission from pig to dog, or vice versa, was not identified (49). In California, United States, feral swine were surveyed for antibodies against *C. burnetii,* revealing that 50% were positive (50). Taken together, these studies highlight that swine may have a potential role in the transmission and spread of *C. burnetii* globally and that further analysis is needed to understand this interaction. To add to this base of knowledge, we sought to investigate feral swine for exposure to *C. burnetii* in the states of Hawaiʻi and Texas given their differential rates of human Q fever cases.

## RESULTS

### Characteristics of feral swine samples from Hawaiʻi and Texas

Serum samples from feral swine were collected between October 2018 and September 2023 by Wildlife Services, part of the United States Department of Agriculture, Animal and Plant Health Inspection Service. Feral swine are routinely removed to protect agriculture, private property, natural resources, human health, and human safety. Samples from a subset of removed animals are collected for national disease surveillance and archival storage. All available sera from the states of Hawaiʻi and Texas between the specified dates were analyzed in the present study to determine *C. burnetii* seroprevalence in feral swine. Samples from the state of Hawaiʻi included 1,233 samples from three counties. The majority of sera samples were from Honolulu County (1,075; 87.19%), and the remaining sera samples were from Maui County (118; 9.57%) and Hawaiʻi County (40; 3.24%). Of the sera from the state of Hawaiʻi, 572 (46.39%) were from female pigs, 647 (52.47%) were from male pigs, and 14 (1.14%) were not reported. The swine samples from Hawaiʻi fall into three age classes, adult (842; 68.29%), sub-adult (387; 31.39%), and unknown (4; 0.32%). Feral swine sera from the state of Texas included 1,063 samples from 101 counties. Of these samples, 535 (50.33%) were from female pigs, 526 (49.48%) were from male pigs, and two (0.19%) were not reported. The swine samples from Texas fall into three age classes, adult (948; 89.18%), sub-adult (112; 10.54%), and unknown (3; 0.28%).

### Seroprevalence of *C. burnetii* in feral swine of Hawaiʻi

**Fig 1:**
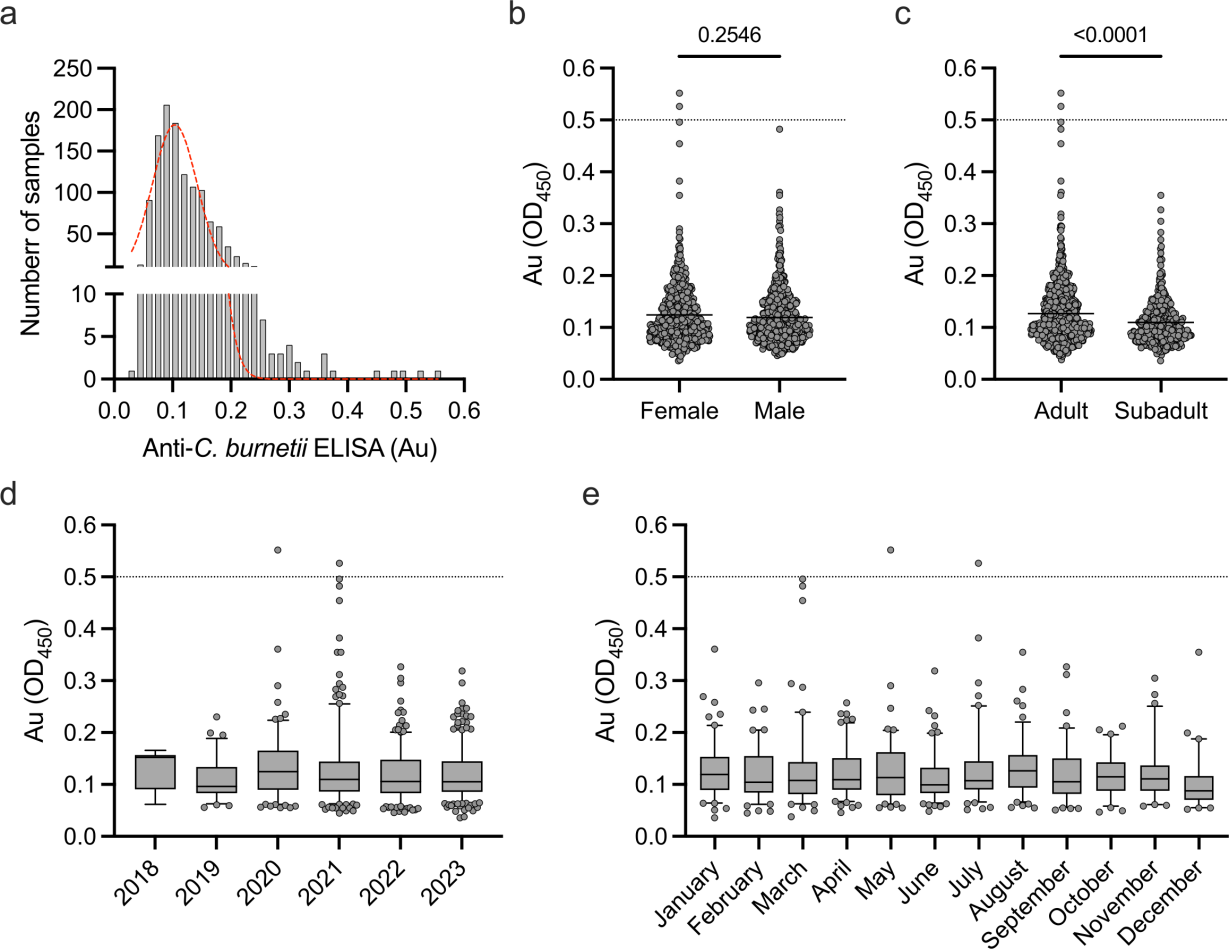
Feral swine in Hawaiʻi have detectable levels of anti-*C. burnetii* antibodies. (**a**) Histogram of 1,233 swine samples from across the state of Hawaiʻi tested via indirect enzyme-linked immunosorbent assay (ELISA) targeting *C. burnetii* antigens. Samples were normalized to a positive control on each individual ELISA plate, and a positive sample is identified when the absorbance (Au) is greater than 0.5. The red line shows frequency distribution across samples tested, and data were determined to follow a non-normal distribution by the Shapiro–Wilk test for normality. (**b**) No significant difference was identified between Au values for female and male pigs by the Mann–Whitney U test. (**c**) Separation by age class shows a significant increase in Au for adult swine by the Mann–Whitney U test. (**d**) Au values separated by year showing positive samples were identified in 2020 and 2021. (**e**) Separation of samples by month of collection identifies that both positive samples were found in the months of May and July. Dotted lines at Au = 0.5 indicate positivity cutoff. Box plots show the interquartile range with whiskers representing the 5th and 95th percentiles, and dots are individual samples that fall outside of those ranges.

**Fig 2:**
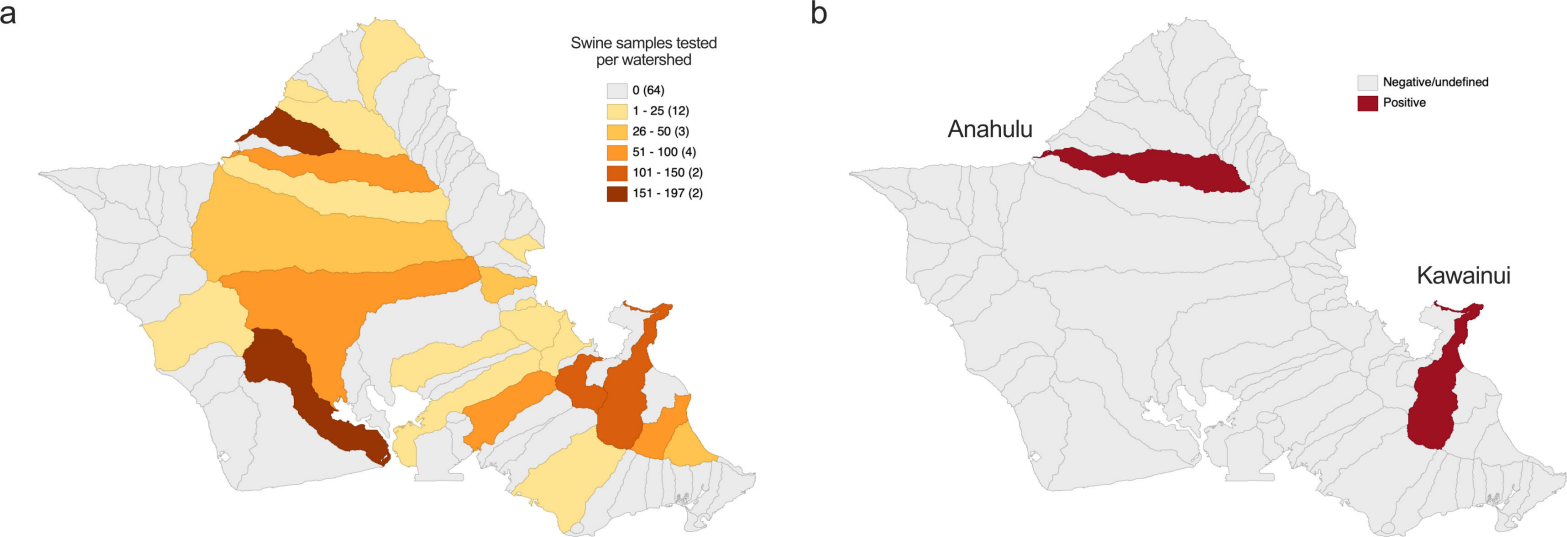
Geographic distribution of feral swine samples and positive serum samples on the island of Oʻahu. (**a**) Distribution of feral swine samples across watershed units on the island of Oʻahu. (**b**) Feral swine samples from watershed units Anahulu and Kawainui were positive for anti-*C. burnetii* antibodies.

The state of Hawaiʻi has a low rate of human Q fever cases but a high population of feral swine relative to land mass (51). In the present study, we sought to investigate the seroprevalence of *C. burnetii* with the hypothesis that rates would be low in Hawaiʻi when compared to states that have higher human Q fever incidence. The feral swine populations in Hawaiʻi that were tested for *C. burnetii* seroprevalence showed a range of absorbances (Au) from 0.036 to 0.552 (µ = 0.121, σ = 0.054) that follow a non-normal distribution (*P* < 0.0001 via Shapiro–Wilk test for normality, Fig. 1a). The vast majority of samples were negative (1,231; 99.84%), but two samples tested (2/1,233; 0.16%) positive (positivity threshold Au ≥0.5), indicating potential exposure to *C. burnetii* (Fig. 1b). Both positive samples were adult female pigs, but analysis of gender across absorbance values showed no significant difference (*P* = 0.2546 via Mann–Whitney U test) between female (µ = 0.124, σ = 0.059) and male (µ = 0.119, σ = 0.050) (Fig. 1b). Comparisons between adult (µ = 0.127, σ = 0.058) and sub-adult (µ = 0.110, σ = 0.044) populations showed a significant increase (*P* < 0.0001 via Mann–Whitney U test) in the absorbance of the older pigs (Fig. 1c). The two positive samples were identified in swine sampled in 2020 and 2021 in May and June, respectively (Fig. 1d and e). We divided the swine sera samples based on spatial locations throughout the state of Hawaiʻi for further analysis. To increase the resolution of this analysis, samples were separated by watershed units rather than county and we focused the spatial mapping on the island of Oʻahu where the majority of samples were collected (1,075; 87.19%), and both positive samples were identified. Samples were collected from 23 different watershed units on the island of Oʻahu, ranging from one to 197 samples per watershed unit (Fig. 2a). Positive samples were found in the Kawainui and Anahulu watersheds along the east and north shores of Oʻahu, respectively (Fig. 2b). Raw seropositivity rates for these watersheds were 0.89% and 1.64%, respectively. We calculated smoothed rates using empirical Bayesian smoothing (EBS) to account for sample size bias, and the seropositivity rate for each watershed was reduced to 0.19%. Overall, feral swine in the state of Hawaiʻi show a low seropositivity rate for *C. burnetii* antibodies.

### Seroprevalence of *C. burnetii* in feral swine of Texas

**Fig 3:**
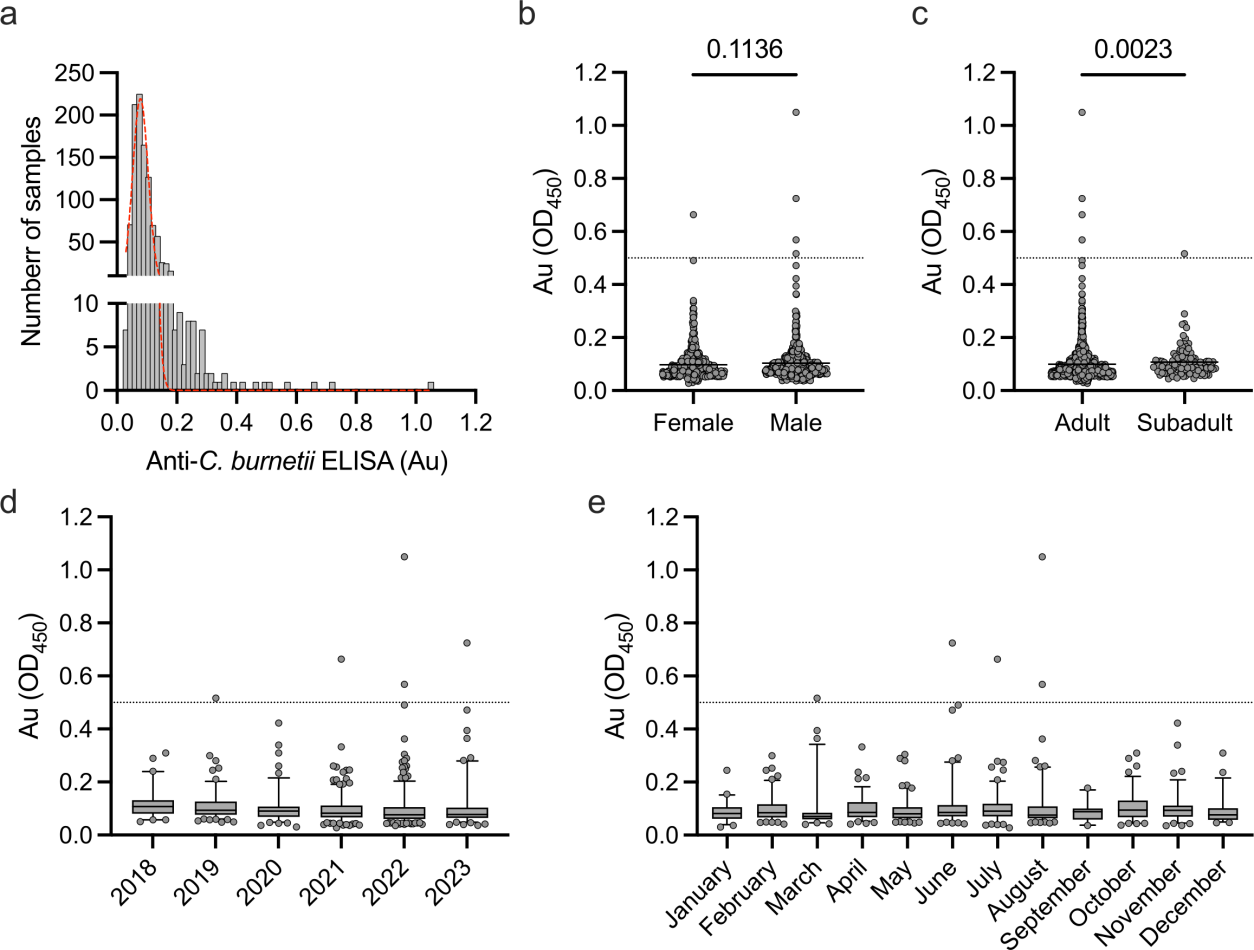
Feral swine in Texas have detectable levels of anti-*C. burnetii* antibodies. (**a**) Histogram of 1,063 swine samples from across the state of Texas tested via indirect ELISA targeting *C. burnetii* antigens. Samples were normalized to a positive control on each individual ELISA plate, and a positive sample is identified when the absorbance (Au) is greater than 0.5. The red line shows frequency distribution across samples tested, and data were determined to follow a non-normal distribution by the Shapiro–Wilk test for normality. (**b**) No significant difference was identified between Au values for female and male pigs by the Mann–Whitney U test. **(c**) There was a significant difference in Au between age classes tested by the Mann–Whitney U test. (**d**) Au values separated by year showing positive samples were identified in 2019, 2021, 2022, and 2023. (**e**) Separation of samples by month of collection identifies positive samples were found in the months of March, June, July, and August. Dotted lines at Au = 0.5 indicate positivity cutoff. Box plots show the interquartile range with whiskers representing the 5th and 95th percentiles, and dots are individual samples that fall outside of those ranges.

The state of Texas accounts for many cases of Q fever throughout the United States, in contrast to the state of Hawaiʻi (52). The high rate of human Q fever cases coupled with the high population of feral swine in Texas compelled the determination of *C. burnetii* seroprevalence rates in that animal population. The feral swine populations in Texas that were tested against *C. burnetii* antigens showed a range of absorbances from 0.028 to 1.050 (µ = 0.100, σ = 0.068) that follow a non-normal distribution (*P* < 0.0001 via Shapiro–Wilk test for normality, Fig. 3a). Five samples (5/1,063; 0.47%) showed an absorbance above the positive threshold (Au ≥0.5), while 1,058 samples (1,058/1,063; 99.53%) were below the threshold (Fig. 3a). Four of the positive samples were from male pigs, and one of the positive samples was from a female pig (Fig. 3b). There was no statistical difference (*P* = 0.1136 via Mann–Whitney U test) between male (µ = 0.104, σ = 0.077) and female (µ = 0.097, σ = 0.057) pigs in Texas (Fig. 3b). Four of the positive samples were adult, and one positive sample was categorized as subadult (Fig. 3c). On the macro level, comparisons between adult (µ = 0.099, σ = 0.069) and sub-adult (µ = 0.108, σ = 0.060) populations showed a significant difference (*P* < 0.0023 via Mann–Whitney U test) in the absorbance between the different age classes (Fig. 3c). Positive samples were identified in the years of 2019, 2021, 2022, and 2023 (Fig. 3d). Seasonally, the majority of positive samples occurred during the months of June, July, and August, with the remaining positive sample being identified in March (Fig. 3e).

**Fig 4:**
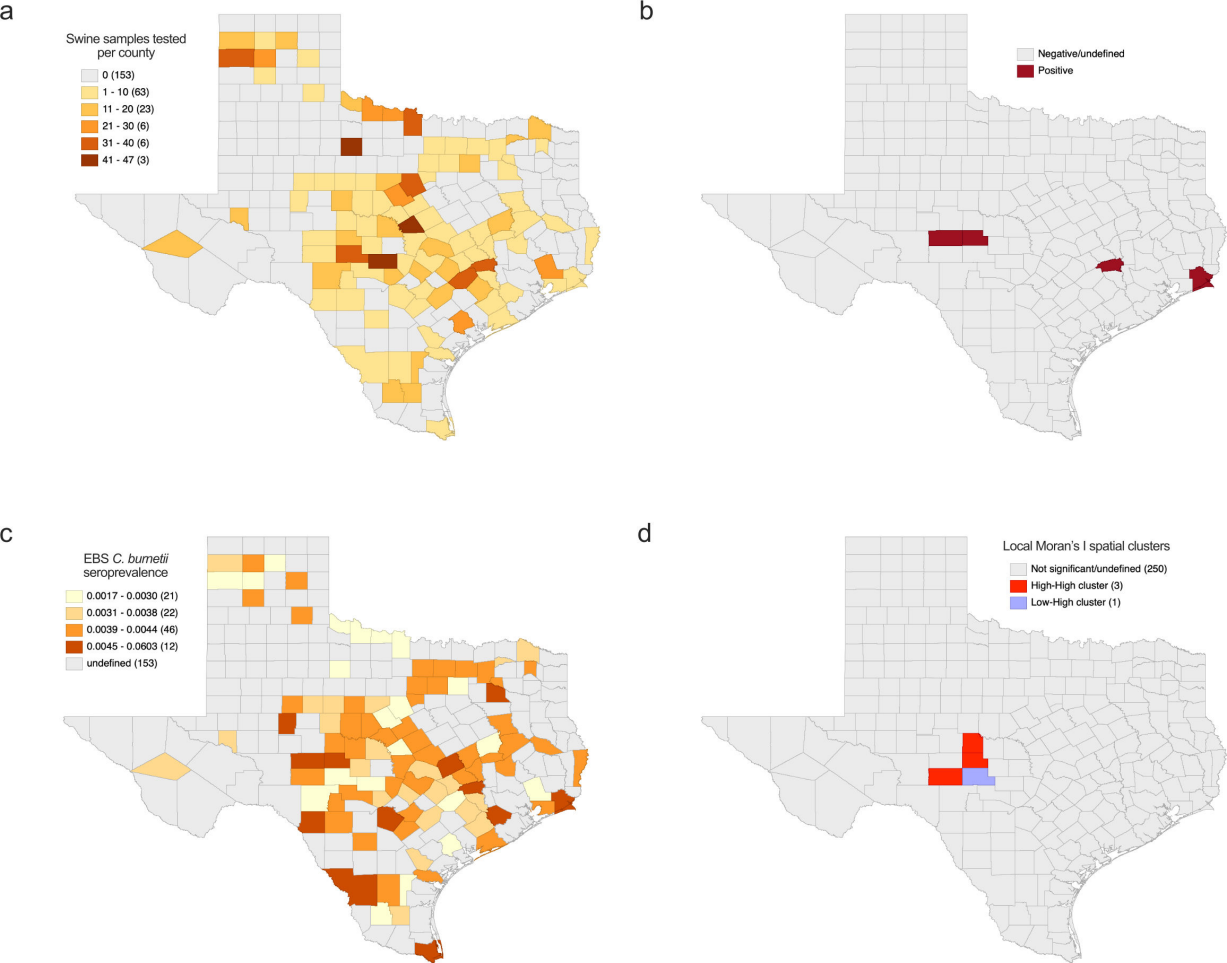
Geographic distribution of *C. burnetii* seroprevalence in the feral swine of Texas**.** (**a**) Distribution of feral swine samples across counties of the state of Texas. **(b**) Feral swine tested positive for anti-*C. burnetii* antibodies in Schleicher, Washington, Jefferson, and Menard counties. (**c**) Empirical Bayesian smoothing (EBS) was used to generate seroprevalence rates for feral swine across the state of Texas. (**d**) Local Moran’s I was used to identify high–high clusters around Concho, Menard, and Sutton counties and a low–high spatial outlier in Kimble County. A description of local Moran’s I spatial clusters is included in Materials and Methods.

To better understand the spatial associations of *C. burnetii* seroprevalence in feral swine across Texas, we separated samples by county. Feral swine sera samples were collected from 101 counties across Texas, and sample sizes per county ranged from one to 47 samples (Fig. 4a). Positive samples were found in Schleicher (Au = 1.05 and Au = 0.57), Washington (Au = 0.72), Jefferson (Au = 0.66), and Menard (Au = 0.52) counties (Fig. 4b). Raw seropositivity rates at the county level were as high as 22.2%, which is likely an overestimate of the actual rate. Therefore, we employed empirical Bayesian smoothing (EBS) to account for sample size bias in the data set to estimate seroprevalence rates across counties that were sampled. We mapped EBS rates across the 101 counties in which samples were tested to generate a risk-associated map using the positivity cutoff described previously (Au ≥0.5) (Fig. 4c). All samples with an absorbance below the cutoff were considered negative. The smoothed rates show that Bexar, Cameron, Fort Bend, Jefferson, Kinney, Menard, Milam, Schleicher, Sterling, Van Zandt, Washington, and Webb counties have the highest predicted *C. burnetii* seroprevalence in the state of Texas, ranging from 0.45% to 6.03% positivity (Fig. 4c). We utilized local spatial autocorrelation via Local Moran’s I to identify any spatial clusters associated with smoothed *C. burnetii* seroprevalence rates of feral swine in Texas. This analysis identified a high–high cluster centralized around Concho, Menard, and Sutton counties and a connected low–high spatial outlier in Kimble County (Fig. 4d).

### Distribution of human Q fever cases in Texas from 2008 to 2022

**Fig 5:**
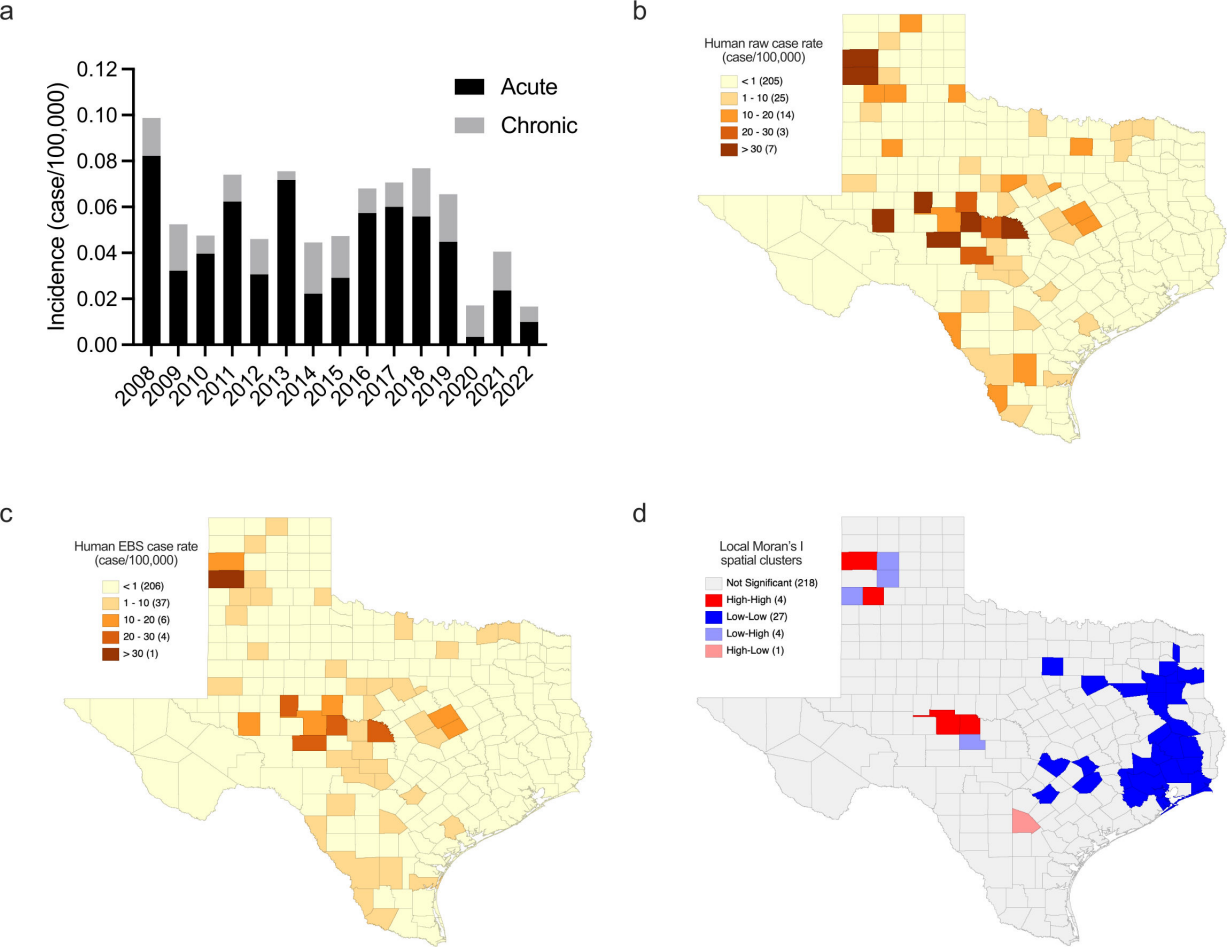
Human Q fever cases from 2008 to 2022**.** (**a**) Reported incidence of human Q fever cases in the state of Texas from 2008 to 2022. Acute and chronic Q fever are differentiated by black and gray boxes, respectively. (**b**) Raw case rate of reported human Q fever per county, excluding cases with unknown areas of disease acquisition. (**c**) EBS case rates of reported human Q fever per county, excluding cases with unknown areas of disease acquisition. (**d**) Local Moran’s I was used to identify high–high clusters around Castro, Concho, Lampasas, and Tom Green counties. Low–low clusters were identified in 27 counties in east and central Texas. Low–high spatial outliers were identified in Menard, Parmer, Potter, and Randall counties, while a high–low spatial outlier was identified in Atascosa County. A description of local Moran’s I spatial clusters is included in Materials and Methods.

Human Q fever is a notable disease in the state of Texas with a requirement to report within 1 day of diagnosis. There were 227 reported cases of human Q fever from 2008 to 2022 with a varied incidence of acute and chronic cases each year (Fig. 5a). Q fever cases were reported in 83 counties throughout the state of Texas, ranging from one to 21 cases. One hundred and forty-four of these cases had epidemiological information on area of disease acquisition used for mapping and comparison to *C. burnetii* seroprevalence in feral swine. We mapped the raw case rate per 100,000 population and identified that seven counties showed greater incidence of Q fever in humans (Fig. 5b). We used empirical Bayesian smoothing (EBS) to account for population variance between counties and identified that Deaf Smith County had an incidence rate of 51.28 cases per 100,000 population (Fig. 5c). Concho, San Saba, and Schleicher counties had the next highest EBS case rates, ranging from 20.66 to 25.71 cases per 100,000 population. Falls, McLennan, Oldham, Runnels, Tom Green, and Upton counties had EBS cases rates per 100,000 population ranging from 10.49 to 13.46. We utilized local spatial autocorrelation via Local Moran’s I to identify any spatial clusters associated with EBS rates of human Q fever in Texas. This analysis identified a high–high cluster around Castro, Concho, Oldham, and Tom Green counties (Fig. 5d). Twenty-seven counties in the eastern portion of the state were identified as low–low clusters of human Q fever incidence (Fig. 5d). Atascosa County was identified as a high–low spatial outlier, and Menard, Parmer, Potter, and Randall counties were identified as low–high spatial outliers (Fig. 5d).

## DISCUSSION

In the current investigation, we selected two ecologically and geographically distinct locations to approach the question of how feral swine may contribute as a potential reservoir of *C. burnetii*. In addition, we wanted to understand how the seroprevalence in feral swine correlates with human Q fever cases to identify any potential links. Hawaiʻi is geographically isolated and lacks reported cases of human Q fever, while having a high population of feral swine (36, 52). Texas, on the other hand, has a higher incidence of human Q fever and a high population of feral swine (36, 52). Comparing the seropositivity rates of feral swine in these states gives us interesting insights. For instance, two swine tested in Hawaiʻi, from two distant watershed units on the populated island of Oʻahu, were positive for exposure to *C. burnetii*. While lack of human Q fever cases in Hawaiʻi can be reasonably linked to reduced occupational risk, the presence of seropositive feral swine suggests that *C. burnetii* may be present on Oʻahu. This could indicate that the zoonotic cycle of *C. burnetii* on the island of Oʻahu is occurring, although a more detailed survey and analysis is needed to confirm this hypothesis.

We found that feral swine in Texas have serological indications of exposure to *C. burnetii*. Positive samples were detected in both male and female swine. The positive samples were identified in sera collected between March and August, mimicking the seasonality observed in human Q fever cases (53). Smoothed county-level seropositivity rates were as high as 6.03%, suggesting that feral swine cannot be ruled out as a contributor to the transmission and spread of *C. burnetii* in the environment and potentially to humans, livestock, or other animals. These results are comparable to those of other global studies of *C. burnetii* seroprevalence in domesticated and feral swine, which reported positivity rates ranging from 0.8% to 14.6% (43–48). In addition to this, we identified a cluster of counties having higher seroprevalence rates when compared to the mean rate across the state. This high–high spatial cluster is centered around Concho, Menard, and Sutton counties and indicates that these counties and their spatial neighbors have higher seroprevalence rates than other counties in Texas. A low–high spatial outlier was also identified in Kimble County that has a lower seroprevalence of *C. burnetii* in feral swine but has spatial neighbors, including the high–high cluster, with higher rates. This analysis indicates that contact with feral swine in these counties could lead to a higher risk for exposure to *C. burnetii* or a potential mechanism of spread from high prevalence to low prevalence regions.

Human cases of Q fever in Texas occur annually, but the reported cases likely do not represent the true extent of the disease burden because approximately 60% of cases are asymptomatic (3). Analysis of reported cases of human Q fever in Texas between the years of 2008 and 2022 identified raw incidence rates for the entire state ranging from 0.017 to 0.099 cases per 100,000 population. Further geographic analysis of cases with disease acquisition information shows that raw county-level incidence rates are as high as 145.77 cases per 100,000 population. To account for variations in population, smoothing shows that incidence rates are as high as 51.28 cases per 100,000 population at the county level. Concho, Deaf Smith, San Saba, and Schleicher counties have the highest smoothed rates of human Q fever in the state. Analysis of smoothed rates across the state of Texas identified a cluster of counties in the eastern portion of the state having lower levels of human Q fever. This analysis indicates that there is a lower chance of exposure to *C. burnetii* in these counties. Atascosa, Menard, Parmer, Potter, and Randall counties were identified as spatial outliers that show rates that are higher or lower than those of their neighbors to a significant degree. Three high–high clusters, where counties and neighboring counties have higher relative disease rates when compared to the overall mean, were also identified and centered around Castro, Concho, Oldham, and Tom Green counties. These high–high clusters indicate that there is a higher risk for exposure to *C. burnetii* when compared to other counties in the state.

Comparison of the *C. burnetii* seroprevalence in feral swine to human Q fever cases shows that there is spatial overlap. In Concho County and neighboring counties, feral swine are more likely to be seropositive to *C. burnetii.* Humans in Concho County and neighboring counties also have a higher chance of *C. burnetii* infection compared to those in the state as a whole. Exposure to *C. burnetii* from contaminated birth products, urine, feces, or milk are likely a common source of infection for both humans and feral swine, but additional research is needed to confirm this hypothesis (2, 25, 26). While contact with infected animals or their excreted products is a source of infection for humans, feral swine also could be infected through scavenging of infected materials or preying on infected livestock. These activities have been observed in feral swine and are potential mechanisms of disease transmission and spread (41, 54, 55). Finally, because *C. burnetii* is also highly resistant to environmental stresses, it can survive for long periods of time and has the potential to become aerosolized, creating another potential source of transmission to feral swine. We suspect that the route of transmission to humans and feral swine may be similar due to geographic overlap with inventories of sheep, goat, and cattle, the main reservoirs for infection. The highest density of domesticated sheep and goats in Texas is in the western region, including Concho County, according to the National Agricultural Statistics Service database (56). Seropositivity rates in goats and sheep have been reported to be 14.5% and 2.7% across the United States, respectively, but to our knowledge, there are no published data on seropositivity rates in these populations of Texas (57, 58). This supports the hypothesis of a common source of transmission from goats and/or sheep to feral swine and humans in this region. Together, these results indicate that feral swine may be a valuable biosentinel and possibly a reservoir with a potential role in transmission and spread of *C. burnetii*. Additional studies to investigate shedding of *C. burnetii* from infected swine are clearly indicated.

Although our research shows that feral swine are exposed to *C. burnetii* and that seroprevalence rates partially overlap with human Q fever incidence in Texas, this study is not without limitations. We were only able to test feral swine sera from 23 watersheds on Oʻahu and 101 counties in Texas. Additionally, our positivity rates in feral swine could be an underestimate because we used a strict cutoff definition for designating samples as a positive sample. Some feral swine showed higher titers of anti-*C. burnetii* antibodies but did not qualify as a positive sample. These swine may have been exposed but could have waning antibody titers due to the timing of the potential infection relative to when the sample was taken. Finally, a potential for cross-reactions with off-target species like *Bartonella*, *Chlamydia*, *Rickettsiae*, and *Legionella* should be considered, although these antibodies are typically present in low titers, making misdiagnosis unlikely (27). Furthermore, the commercially available kit used in this study has been extensively used in the literature (43–46, 59–61). Overall, in this study, we show that feral swine have the potential to be a reservoir for the spread and transmission of *C. burnetii* to humans, livestock, and other animals. Additional investigation into other regions with feral swine, beyond the scope of this work, will aid in obtaining a more complete understanding of *C. burnetii-*associated risk. Finally, a further increase in the feral swine population and geographic spread across the United States will compound the risks associated with *C. burnetii* distribution and infection.

## MATERIALS AND METHODS

### Sample acquisition

The National Feral Swine Damage Management Program (NFSP) within the Animal and Plant Health Inspection Service of the USDA was established to help protect agricultural resources, natural resources, property, human health, and human safety through the management of invasive feral swine in the United States and its territories. The NFSP aims to monitor feral swine for pathogens that can affect human and animal health. During routine feral swine removal efforts, field personnel collect serum samples and metadata for national surveillance efforts and retrospective disease surveillance and disease risk analysis (41). Samples collected between October 2018 and September 2023 from the states of Hawaiʻi and Texas (*n* = 2,308) were obtained through a partnership with the NFSP. Postmortem blood was collected from male and female swine, and serum was extracted within 12 hours of clotting. Field personnel estimated the age and classified each sample into two age class categories, adult (≥1 year of age) or subadult (<1 year of age). Serum was shipped to the National Wildlife Research Center in Fort Collins, CO, USA, and stored at −80°C until testing.

### Serological testing and analysis

An indirect ELISA was used to determine *C. burnetii* seroprevalence of feral swine in Hawaiʻi and Texas. All samples were screened in duplicate using the ID Screen Q Fever Indirect Multi-species ELISA kit (Innovative Diagnostics, France) following the manufacturer’s instructions. This indirect ELISA contains phase I and phase II *C. burnetii* antigens from an aborted bovine placenta and positive bovine serum control that has been used to test serum from swine previously (43–46). The absorbance cutoffs published by the manufacturer indicate that any samples between 50% and 80% of the positive control are considered positive and samples greater than 80% of the positive control are strong positive. We used the recommended 50% cutoff to determine positive results in this study, as has been done in other work investigating seroprevalence in swine populations (43–46). Briefly, the mean raw absorbance at 450 nm (Au) of each sample was normalized to a positive control on each individual plate (Au = 1.0). Samples with a mean normalized Au above 0.5 were considered positive, with all other samples below this cutoff considered negative. Forty-four percent of all samples (including all positive samples) were retested to ensure accurate results. Normalized Au values were collated in Microsoft Excel version 16.78 (Microsoft Corporation, Washington, USA), and data were visualized and statistically analyzed with GraphPad Prism version 9.5.1 (San Diego, California, USA). Statistical analysis of Au values was carried out as described in the text.

### Mapping seroprevalence rates

Seroprevalence was mapped across the states of Hawaiʻi and Texas to further analyze *C. burnetii* risk associated with feral swine. Samples were separated by county in Texas to determine spatial variations in seroprevalence. In Hawaiʻi, samples were separated by the watershed unit to give higher spatial resolution. County-level GIS data used by the Texas Department of Transportation (https://gis-txdot.opendata.arcgis.com) were downloaded on 29 October 2023 and used to map rates in the state. Watershed level GIS data from the state of Hawaiʻi were downloaded from Hawaiʻi Statewide GIS Program (https://geoportal.hawaii.gov/) on 4 December 2023 and used to generate maps. Raw seroprevalence rates were determined using the number of positive samples and the number of total samples from each spatial unit. Due to variations in sample size from each spatial unit, we used empirical Bayesian smoothing (EBS) to adjust prevalence rates as previously described (62, 63). Raw and EBS rates were calculated and mapped in GeoDa version 1.22.

### Local spatial autocorrelation of seroprevalence

The local Moran’s I statistic is used to identify spatial hotspots (high–high clusters), spatial cold spots (low–low clusters), or spatial outliers (high–low or low–high clusters) within a given data set and is previously described elsewhere (64). Briefly, the local Moran’s I statistic is designed to reject the null hypotheses of spatial randomness and uses computational permutations to determine a pseudo-*P*-value. Significant clusters indicate that the spatial unit and its neighbors fall into one of four categories. A high–high cluster indicates that the spatial unit indicated and its neighbors have similarly high values compared to the mean of all spatial units. A low–low cluster indicates that the spatial unit and its neighbors have similarly low values compared to the mean of all spatial units. A high–low spatial outlier has a higher value compared to its neighbors that are low, and a low–high spatial outlier has a low value compared to its neighbors that are high. We employed this analysis to determine if there are any regions of higher or lower risk associated with feral swine and exposure to *C. burnetii*. EBS rates were used to calculate the local Moran’s I statistic in GeoDa version 1.22, as previously described (62). We used a first-order queen contiguity and 99,999 computational permutations to determine the pseudo-*P*-value. Any spatial units with no neighbors were excluded from the analysis. A cutoff value of *P* < 0.01 was used to determine statistical significance of any identified clusters.

### Acquisition and analysis of human Q fever case counts from 2008-2022

Human Q fever case counts at the county level in the state of Texas were requested and provided by the Zoonosis Control Branch of the Texas Department of State Health Services. This epidemiological information contains no personal identifiable information and is used for statistical purposes only to identify counties associated with a higher risk of *C. burnetii* infection. To understand the spatial distribution of human Q fever in Texas, acute and chronic case counts were combined, and the resulting maps produced represent the extent of reported human Q fever cases from 2008 to 2022 that have epidemiological information on the area of disease acquisition. Raw and EBS case rates for human Q fever were calculated using GeoDa version 1.22, as described previously. Human Q fever EBS rates were used to calculate the local Moran’s I statistic to identify significant spatial clusters of counties, as described previously.
